# The Assessment of Brain Volume Differences in Idiopathic Central Precocious Puberty Girls; Comparison of Age-Matched Girls and Normal Puberty Girls

**DOI:** 10.3390/children8090797

**Published:** 2021-09-11

**Authors:** Shin-Eui Park, Ji-Ye Ahn, Eun-Young Kim

**Affiliations:** 1 Advanced Institute of Aging Science, Chonnam National University, Gwangju 61453, Korea; shineuipark@gmail.com; 2 Department of Pediatrics, Chosun University Hospital, Gwangju 61453, Korea; sskey@chosun.ac.kr

**Keywords:** precocious puberty, luteinizing hormone, magnetic resonance imaging, gray matter, white matter

## Abstract

Objective: Although there have been several studies on the neuroanatomical changes in idiopathic central precocious puberty (ICPP), the association between each brain region and ICPP has not yet been clearly elucidated. This study aimed to evaluate the difference in brain structure in ICPP compared with age-matched healthy controls and normal puberty controls, and additionally the correlation between brain volume difference and the luteinizing hormone (LH). Materials and Methods: The study enrolled fifteen girls with ICPP, as well as 15 age-matched healthy girls and 15 normal puberty girls as controls. The subjects underwent a 1.5 Tesla Avanto MR Scanner. Anatomical T1-weighted images were acquired with a T1 spin-echo sequence. The volumes of total and regional brain were compared with each of the two control groups and analyzed through the paired T-test, and the brain region related to the peak LH level was also analyzed through a simple correlation test. Results: The mean age of the ICPP group, age-matched group, and puberty group were 8.0 ± 0.9 years, 7.8 ± 0.9 years, and 11.9 ± 0.9 years, respectively. In our findings, the regional cerebral volumes in ICPP were different from age-matched controls. Compared with controls, ICPP showed a significant increase in gray matter (GM) volumes (the medial prefrontal cortex, superior parietal gyrus, supramarginal gyrus, angular gyrus, postcentral gyrus, superior occipital gyrus, cuneus, hippocampus, parahippocampal gyrus, posterior cingulate gyrus (PCgG), cerebellar cortex (Cb)) and in white matter (WM) volumes (the insular, caudate, splenium of corpus callosum (*p* < 0.001)). Especially, the GM volumes of the PCgG (r = 0.57, *p* = 0.03) and Cb (r = 0.53, *p* = 0.04) were correlated positively with LH concentrations stimulated by the gonadotropin-releasing hormone agonist. Compared to the normal puberty control, no significant difference in GM volume was found. Conclusions: This study showed the overall brain volumetric differences between ICPP girls and age-matched controls using voxel-based morphometric analysis, and further showed the correlation between brain volume and the sex hormone in ICPP. Through a comparison between the two groups, the cerebral development pattern of ICPP is similar to that of normal puberty, and these local differences in cerebral volume may affect social and congenital changes. These findings will be useful for understanding the neuroanatomical mechanisms on the specific morphological variations associated with ICPP.

## 1. Introduction

Precocious puberty (PP) is defined as the development of secondary sexual characteristics before the age of 8 years in girls and 9 years in boys [1]. In 1999, the American Academy of Pediatrics presented a proposal to classify a case of a white girl before the age of 7 years and a black girl before age of 6 years [2] based on the Pediatric Research in Office Setting (PROS) report, which was published in the United States in 1997, diagnosing the secondary growth of about 17,000 healthy young children between the ages of 3 to 12 years [3]. Although there is no consensus, the onset of PP is normally defined as Tanner stage 2 in girls before the age of eight years and in boys before the age of 9 years [1]. Precocious puberty can cause many problems, such as pressure on different body types of the same age, confusion caused by the mismatch between mental maturity and physical maturity, and short stature caused by premature closing of the growth plate. Furthermore, early maturation is believed to have a negative psychological impact on girls [4,5], and girls with early pubertal timing are more vulnerable to stressors [6]. For these reasons, social interest in PP is growing progressively. PP could be classified as gonadotropin dependent. In the case of the hypothalamic-pituitary-gonadal (HPG) axis active in PP, it is called true/central precocious puberty (CPP). Otherwise, it is called pseudo/peripheral precocious puberty [7,8]. The etiologies of CPP can be classified as organic or idiopathic by whether the abnormality of the central nervous system stimulating HPG is accompanied. Idiopathic CPP (ICPP) accounts for 80–95% in girls and 30–40% in boys, relatively more related to girls [2]. So far, it is known that genetic factors, nutritional status and obesity, environmental hormones, and stress are involved in the causes of ICPP [9,10,11]. As a treatment for this, Gonadotropin-releasing hormone agonists (GnRHa) are used to sensitize GnRH receptors to decrease receptor sensitivity and down-regulate receptor expression to suppress gonadotropin secretion [7,8,12].

Currently, the physical developmental and endocrinological mechanisms have be-come known in ICPP, and there are several studies that examine with great interest the changes in brain structure and function that occur with the progression of puberty [13,14]. However, to our knowledge, there are no studies on brain structure comparing normal and early puberty in the same age group and comparing different age groups with similar puberty.

The purpose of our study was to compare the cerebral volume differences between ICPP girls, age-matched girls, and normal puberty girls using voxel-based morphometry (VBM), which is a method to quantify the local volume change in the cerebral cortex objectively, and, additionally, to evaluate the influence of the luteinizing hormone (LH) on brain structure.

## 2. Materials and Methods

### 2.1. Subjects

The present study included fifteen girls with ICPP, as well as 15 age-matched healthy and normal puberty groups. The age of subjects was seven to twelve. All participants are recruited by the Department of Pediatrics at Chosun University Hospital. The ICPP was diagnosed by a pediatrician in accordance with the following criteria as in a previous study [15]: first, objective breast budding appearing before the age of eight years; second, if advanced bone age (BA) is more than chronological age; third, LH stimulated by GnRHa ≥ 5.0 IU/L. As included in the ICPP diagnosis, all participants included only those with normal magnetic resonance imaging (MRI). In patients with ICPP, BA was measured on the radiographs of the left hand and wrist using the Greulich Pyle method, and LH levels were referenced to the peak levels during the stimulation test at the diagnosis of ICPP. This protocol was approved by the Institutional Review Board of Chosun University Hospital (IRB No. 2013-12-004).

### 2.2. Data Acquisition

Scanning was performed on a 1.5 Tesla Avanto MR Scanner (Siemens Medical Solutions, Erlangen, Germany) with an 8-channel birdcage type of head coil. Anatomical T1-weighted images were acquired with a T1 spin-echo sequence with the following parameters: repetition time (TR)/echo time (TE) = 639/9.1 ms, field-of-view (FOV) = 16 × 16 cm^2^, matrix size = 320 × 320, number of excitation (NEX) = 1 and slice thickness = 2 mm, and 10% gaps.

### 2.3. Postprocessing and Statistical Analysis

The data were post-processed using Statistical Parametric Mapping 8 (SPM8; www.fil.ion.ucl.ac.uk/spm, accessed on 2 March 2015) implemented in MATLAB 7.13 (The Mathworks, Natick, MA, USA). First, the T1-weighted images were aligned with the plane of the anterior and posterior commissure. Field bias correction was performed to correct the nonuniformity field on images. Gray matter, white matter, and the cerebrospinal fluid of each tissue image were segmented using tissue probability maps based on the International Consortium of Brain Mapping space template type of East Asian Brains, as implemented in SPM 8. After visually checking the segmentation image for errors, gray and white matter segmentation is used to create a study-specific template with double deformed anatomical records using the Exponential Lie Algebra (DARTEL) tool. Then, an individual gray matter image is deformed into the template, aligned with the Montreal Neurology Institute (MNI) space, and re-cut into 1.5 mm isotropic voxels. Finally, the voxel values are weighted by the Jacobian to preserve the area volume information, and a full-width 12 mm smoothing kernel is applied at half of the maximum value to increase the signal-to-noise ratio. To illustrate the difference in overall brain size between ICPP patients and the two control groups, the total intracranial volume (TICV) of each participant was calculated by adding the voxel values of gray matter (GM), white matter (WM), and cerebrospinal fluid (CSF), which is divided and used as a covariate in the analysis of covariance. An independent two-sample t-test was established to seek areas with different volumes for GM and WM. In order to control multiple comparisons, the *p*-value was adjusted to *p* < 0.001 uncorrected, excluding 100 voxels. Then, we used the MRI-cron software (www.psychology.nottingham.ac.uk/staff/cr1/mricron.html, accessed on 29 March 2015) to perform anatomical labeling of specific areas of volume changes between precocious puberty patients and healthy controls, which helps to identify changes. The x, y, and z coordinates of the maximum t value are based on the MNI brain space. SPSS 19.0 (SPSS Inc., Chicago, IL, USA) was used to analyze the correlation between brain volume and the LH peak level through a simple correlation test.

## 3. Results

### 3.1. Clinical Characteristics

The mean age of 15 ICPP girls was 8.0 ± 0.8 years old, and the mean age of age-matched healthy girls was 7.8 ± 0.9 years old. The mean age of normal puberty girls was 11.9 ± 0.9 years old, and the 15 girls with ICPP had advanced bone age (10.6 ± 1.0 years) and breast development (Tanner stage 2–4). Peak LH levels stimulated by the GnRH of ICPP girls were 15.9 ± 14.9 mlU/mL.

### 3.2. Total Intracranial Volume (TICV)

The TICV was 1194.6 ± 122.4 ml for ICPP girls, 1139.0 ± 75.5 ml for age-matched controls, and 1173.93 ± 68.93 mL for normal puberty controls. There is no significant difference between the three groups in TICV (Table 1).

### 3.3. Regional Volume Difference

#### 3.3.1. ICPP vs. Age-Matched Control

**Figure 1:**
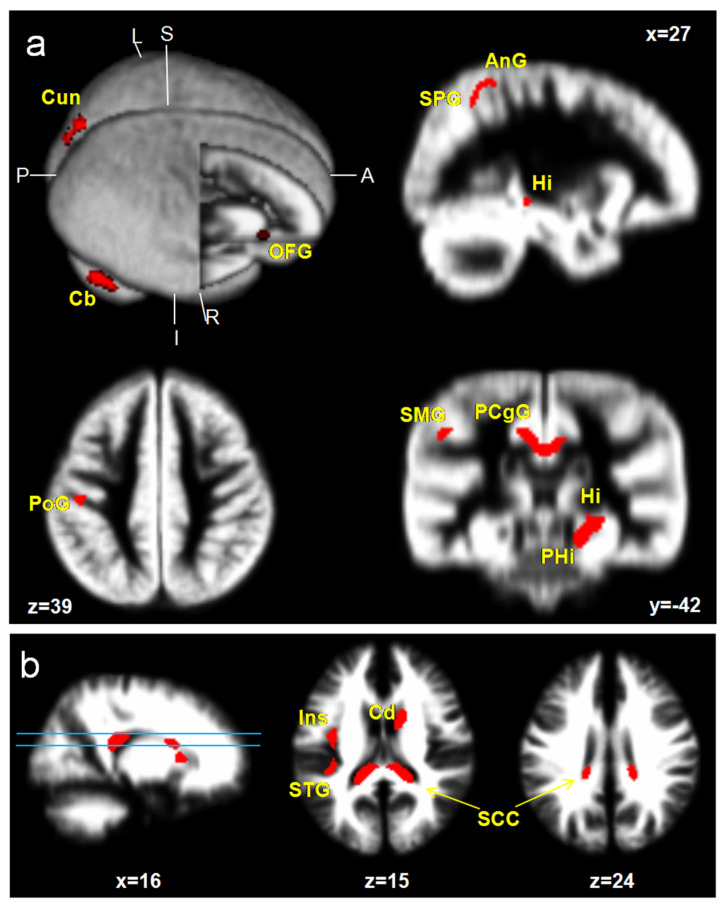
Brain areas with a significant increase in GM (**a**) and WM (**b**) volumes in ICPP girls in contrast to age-matched healthy girls (uncorrected; *p* < 0.001, excluded 100 voxels). Hi, hippocampus; PHG, parahippocampal gyrus; PCgG, posterior cingulate gyrus; Cun, cuneus; PoG, postcentral gyrus; SCC, splenium of corpus callosum; mPFC, medial prefrontal cortex; Ang, angular gyrus; SMG, supramarginal gyrus; SPG, superior parietal gyrus; STG, superior temporal gyrus; Ins, insula; Cd, caudate nucleus; Cb, cerebellar cortex.

ICPP girls showed a significant increase in GM volumes; specifically, the medial prefrontal cortex (mPFC), superior parietal gyrus (SPG), supramarginal gyrus (SMG), angular gyrus (AnG), postcentral gyrus (PoG), superior occipital gyrus (SOG), cuneus (Cun), hippocampus (Hi), parahippocampal gyrus (PHG), posterior cingulate gyrus (PCgG), and cerebellar cortex (Cb) (uncorrected *p* < 0.001, excluded 100 voxels). However, reduced brain areas were not found in ICPP girls. Increased WM volumes are shown at the insula (Ins), caudate nucleus (Cd), and splenium of corpus callosum (SCC) (Figure 1, Table 2).

#### 3.3.2. ICPP vs. Normal Puberty Control

**Figure 2:**
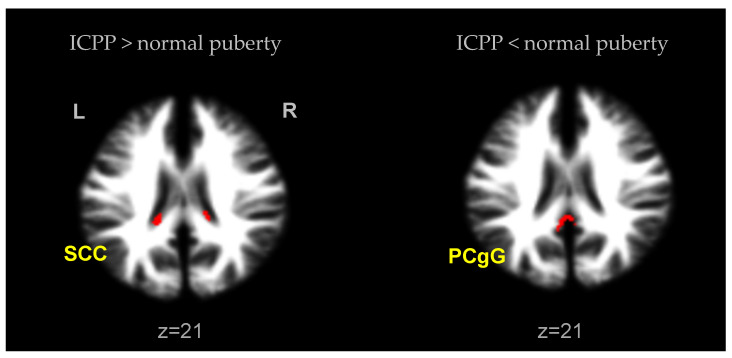
Brain areas with a significant difference in WM volumes in ICPP girls in contrast to normal puberty girls (uncorrected; *p* < 0.001, excluded 100 voxels). SCC, splenium of corpus callosum; PCgG, posterior cingulate gyrus.

ICPP girls showed no significant difference in GM volumes compared to normal puberty controls, but significantly increased WM volume in SCC. Conversely, a statistically significant increase in the volume of PCgG in WM was observed in the normal puberty group compared to the ICPP group (uncorrected *p* < 0.001, excluded 100 voxels) (Figure 2, Table 3).

### 3.4. Correlation of Brain Volume with LH Level

**Figure 3:**
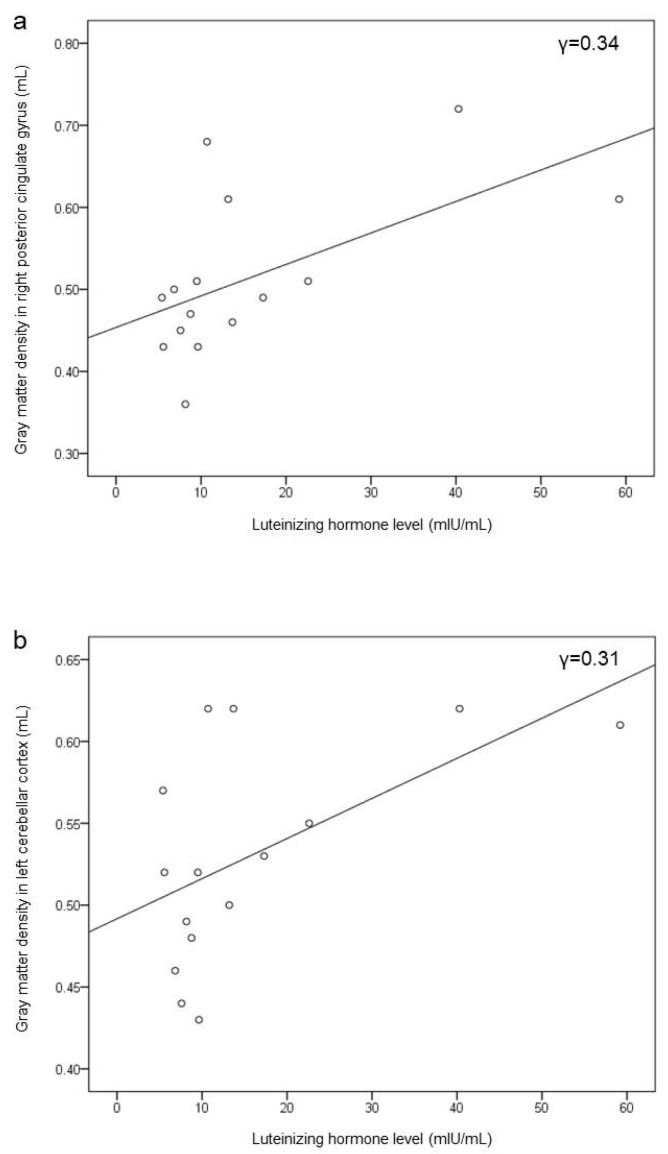
Scatterplot of correlation between LH level and increased GM; PCgG (MNI x, y, and z coordinates 3, −48, and 21) (**a**) and Cb (−23, −69, −47) (**b**) in ICPP girls. Each correlation coefficient is 0.57 (p* = 0.03) and 0.53 (p* = 0.04) in PCgG and Cb. * p value is uncorrected on this model.

Figure 3 shows the correlation between the GM volumes and LH level in ICPP girls. The volumes of the right PCgG and left Cb were correlated positively with the LH level (*r* = 0.57*,* uncorrected *p* = 0.03 and *r* = 0.53, uncorrected *p* = 0.04, respectively).

## 4. Discussion

In this study, the brain’s developmental morphological changes and the influence of the LH on brain structure in ICPP girls were assessed by qualitative and quantitative analyses by using a VBM study based on the DARTEL algorithm and statistical approach. At first, two groups composed of ICPP girls (age 7 to 9) and the age- and sex-matched group were analyzed by a two-sample t-test (*p* < 0.001). The areas with increased GM in ICPP were the frontal lobe: mPFC; parietal lobe: SPG, SMG, AnG, and PoG; occipital lobe: SOG, and Cuneus; limbic system; Hi, PHG, and PCgG; cerebellum: Cb. The increased WM volumes were observed at Ins, Cd, and SCC. However, reduced WM areas were not observed. A longitudinal pediatric neuroimaging study [16] reported that the brain growth patterns across ages 4 to 22 show linear increases in WM, but in GM, they demonstrated quadratic forms in which there was increased GM in previous puberty followed by a decrease post-puberty. Specifically, the developmental curve of the frontal lobe and parietal lobe peaked at about 12 years old, while the developmental curve of the temporal lobe peaked at about 16 years old, while the GM of the occipital lobe continued to increase until the age of 20 years. Our results, specifically, the increased GM and WM volumes in ICPP girls over the age- and sex-matched group, suggest that brain development during PP showed a similar pattern to normal puberty. Thus, it is speculated that ICPP girls may be suffering an onset of synaptic proliferation and reorganization similar to puberty [16].

The prefrontal cortex is involved in planning complex cognitive behavior, expressing personality, decision making, and moderating social behavior [17]. An experimental study [18] using animal models revealed that at early puberty, stressed animals displayed significantly lower densities of neurons in the mPFC compared with age-matched unstressed controls. Another experimental study compared the number of neurons and glia in mPFC after a gonadectomy in rats and showed that female rats who underwent a gonadectomy showed more neurons. Based on these results, it was proved that the volume increased until puberty, and then decreased until adulthood due to the regulation of the ovarian hormone [19]. Unlike the previous two animal experiments, in our results, a decrease in the volume of mPFC was observed in ICPP girls. Although the basis to explain this result is not clear, there is a part that ICPP actually increases the volume by 1–2 years ahead of children of the same age according to bone age, and there is a part that is thought to have an increase in volume. It is presumed to be the difference before receiving. Since most of the children had brain images at the time they were diagnosed, the correlation between the hormonal effect is not clear. It is thought that additional studies, such as a comparison of the estrogen level and volume of mPFC, are needed to examine the relationship clearly.

The parietal cortex is closely related to mental rotation. Mental rotation is the ability to rotate two-dimensional and three-dimensional objects within the human mind. The superior parietal cortex is referred to as an important supportive region for mental rotation [20]. Some studies [21,22] suggested that early pubertal maturation is associated with lower spatial ability: the girls with ICPP performed significantly less well in spatial cognitive tests than sex-, age-matched controls. From the results of the increased GM in the parietal cortex, especially SPG, in this study, it could be speculated that the abnormal development of parietal regions caused by the etiology of PP may lead to dysfunction in spatial cognition.

The SOG and Cun belonging to the occipital lobe were observed as increased GM. The occipital lobe, especially Cun functioning as a visual, is noticeably developed during the puberty period. The Cun is known for its involvement in basic visual processing and emotion [23]. This brain development activity seems to be associated with children’s sensitive response to visual stimuli such as appearance or fashion. Most puberty children have a sensitive curiosity for the opposite sex and internal problems such as emotional issues. Some studies [24,25] have reported that girls with an earlier onset of puberty are concerned about lower self-esteem, a feeling of being unhealthy, loneliness, and depression. A research study [26] reported that adolescents with precocious puberty tend to be earlier in starting their adolescent sex life, though they remain within the normal range of current standards. It is also commonly recognized that children undergoing puberty do not well comply with their parents when their parents are authoritarian or oppressive. These sensitivities for the curiosity of psychosexuality and appearance seem to be associated with brain development.

When puberty children are making a judgment about behavior and other matters, the limbic system is more used than the frontal lobe, relatively. The limbic system is closely related to affective and emotional factors [27,28]. The increased GM, such as the Hi, PHG, and PCgG included in the limbic areas found by this study, could suggest that ICPP girls may be more sensitive to a fear of sexuality, feelings of loneliness, and behavioral problems, with high scores on withdrawal, anxiety/depression, and somatic complaints. In a previous study [29], the girls with premature adrenarche had significantly more oppositional defiant disorder, disruptive behavior disorder, and anxiety symptoms than on-time girls. Another study found that stress during puberty also caused an increase in Hi volume [30].

An interesting feature among our results is that increased PCgG volume had a positive correlation with the LH. Recently, Menk TAS et al. [31] reported that physical and psychological stress on girls with PP might be mediated by treatment with a gonadotropin-releasing hormone agonist (GnRHa). In other words, the stress from prematurity in girls with PP could be associated with the gonadotropin hormone. Summing up the contents of the above, the psychosocial and physical stress related to the different body image comparisons with peers, hormonal changes, and changes in the brain development of limbic regions seem to be closely related to each other.

The cerebellum is histologically connected to the dorsolateral prefrontal cortex, medial frontal cortex, and the parietal and superior temporal areas [32,33], and it plays an important role in higher cognitive function and emotional regulation [34,35]. These advanced cognitive functions dominated by the cerebellum continue to improve during childhood and adolescence, indicating that the cerebellum may be undergoing substantial development during this period [36]. In our study, we observed an increase in the amount of GM and a positive hormonal correlation with the Cb in ICPP.

Increased WM volumes are observed in the SCC, Ins, Cd in ICPP girls. WM composed of myelin surrounding the nerve fibers is the tissue through which messages pass between different areas of GM within the central nervous system. Moreover, these areas are contained within the frontoparietal control network involved in executive control, defined as intrinsic connectivity networks [37,38]. Other studies on the corpus callosum (CC) and puberty also found a positive correlation between CC thickness or density (especially part of the splenium) according to puberty progression [39,40].

To investigate the effect of precocious puberty itself, we also included an analysis of differences from the normal puberty group (age 9 to 12). In both groups, there was no significant difference in the brain volume in gray matter. In white matter, a significant increase in the SCC was observed in ICPP girls compared to the normal group with a similar puberty status. Conversely, in normal puberty girls, a significant increase in the PCgG compared to ICPP girls was found. Regarding these results, we think that because early puberty girls feel more emotional changes to body changes than normal puberty girls, this difference is related to an increase in the volume of the SCC, an area that processes related information [39]. However, the PCgG is also an area related to such emotional processing, and it is difficult to interpret the opposite result. It is thought that a larger sample size is needed for the change in white matter.

Similarly, Chen et al. conducted a study on gray matter (GM) volume and functional connectivity (FC) in association with pubertal hormone levels in precocious puberty girls [14]. In this study, compared with peripheral PP, a decrease in the volume of the left insula and left fusiform gyrus was observed in the central PP girl, which showed a negative correlation with the peak FSH level. The authors explained that this area is responsible for affective, cognitive, regulatory functions and facial recognition, and that premature activation of the HPG axis caused changes in this area. No reduction in insular inclusion gray matter was observed in our study. It is thought that continuous research on hormonal levels and regional brain volume is needed in the future.

According to a previous study on the association between height and GM volume in healthy children, there was a significant positive correlation in the bilateral prefrontal cortex area, temporoparietal region, and cerebellum [41]. These results are also similar to those of our study, which are thought to be due to the fact that most girls with PP are 1.5 years taller than their peers. However, not all PP girls are taller than their peers, so research that considers all aspects is needed to determine accurately whether these changes are due to mental maturity, hormonal changes during puberty, or height.

In summary, increased GM and WM volumes were observed in the whole cerebral area of ICPP girls compared to the age-matched control group. These observed brain regions were related to emotional and cognitive function, and correlated with the sex hormone additionally. This suggests that the cerebral development pattern of ICPP is similar to that of normal puberty, and these local differences in cerebral volume may affect social and congenital changes.

There are several limitations of this study. First, the participants consisted of fifteen ICPP girls and two groups of controls of fifteen girls each, which altogether represent a relatively small sample size, and thus provided lenient statistical power to the data. However, a normality test (KS) was performed for the participants to ensure a normal distribution of data. Second, we controlled only females in this study. The gender-related information cannot be acquired, and bias may have been induced. Third, the 1.5 T MRI scanner and eight channel head coil were used for this study. There is a relatively lower resolution. Fourth, in MRI analysis, the adjusted *p*-value is analyzed as an uncorrected *p* < 0.001, excluding 100 voxels, and there is a possibility of false positives because of other family-wise error or false-discovery rate controls that were not performed. To make up for this challenge, a rigid statistical approach and post-processing were performed.

## 5. Conclusions

This study showed the overall brain volumetric differences between girls with ICPP and controls using VBM analysis, and further showed the correlation between brain volume and the sex hormone in ICPP girls. These findings will be useful for understanding the neuroanatomical mechanisms on the specific morphological variations associated with precocious puberty.

## Figures and Tables

**Table 1:** Total intracranial volume estimates in milliliters.

	Girls with ICPP (N = 15)	Normal Puberty Girls (N = 15)	Age-Matched Girls (N = 15)	Statistical Analysis (*p*-Value)			
				ANOVA	ICPP vs. Puberty	ICPP vs. Age-Matched	Puberty vs. Age-Matched
Gray matter	708.15 ± 61.14	666.16 ± 33.65	670.24 ± 59.64	*p* = 0.364	*p* = 0.331	*p* = 0.727	*p* = 0.780
White matter	486.47 ± 107.97	507.77 ± 64.19	468.75 ± 49.61	*p* = 0.508	*p* = 0.726	*p* = 0.921	*p* = 0.487
Total	1194.63 ± 122.39	1173.93 ± 68.93	1138.99 ± 75.49	*p* = 0.754	*p* = 0.984	*p* = 0.752	*p* = 0.847

**Table 2:** Brain regions with a significant increase in GM and WM volumes in ICPP over age-matched controls (uncorrected; *p* < 0.001, excluded 100 voxels).

Brain Area	Abbr.	Side	BA*	MNI Coordinate			t-Value	Cluster Size (Voxels)
				x	y	z		
*Gray matter*								
Medial prefrontal cortex	mPFC	R	11	21	24	−12	4.48	143
Superior parietal gyrus	SPG	R	40	29	−46	49	5.21	129
Supramarginal gyrus	SMG	L	48	−47	−30	34	4.98	179
Angular gyrus	AnG	R	7	27	−54	46	4.79	129
Postcentral gyrus	PoG	L	3	−44	−22	37	5.45	179
Superior occipital gyrus	SOG	L	19	−6	−82	43	3.96	116
Cuneus	Cun	L	19	−5	−85	34	3.75	116
Hippocampus	Hi	R	37	26	−30	−5	4.12	497
Parahippocampal gyrus	PHG	L	30	−21	−18	−27	3.91	180
Posterior cingulate gyrus	PCgG	L	23	−9	−39	30	5.44	1485
Cerebellar cortex	Cb	L	-	−50	−67	−29	5.69	888
*White matter*								
Insular	Ins	L	48	−33	2	9	4.58	750
Caudate	Cd	R	-	12	2	18	4.35	419
Splenium of corpus callosum	SCC	R	-	5	−28	13	4.88	533

* BA indicates Brodmann’s area.

**Table 3:** Brain regions showing different white matter volumes between patients with ICPP and normal puberty controls (uncorrected; *p* < 0.001, excluded 100 voxels).

Brain Area	Abbr.	Side	BA*	MNI Coordinate			t-Value	Cluster Size (Voxels)
				x	y	z		
*ICPP*								
Splenium of corpus callosum	SCC	R	-	15	−30	19	4.20	160
Splenium of corpus callosum	SCC	L	-	−15	−34	18	4.65	194
*Normal puberty*								
Posterior cingulate gyrus	PCgG	L	26	−3	−43	21	4.60	172

*BA indicates Brodmann’s area.

## Data Availability

The data presented in this study are available upon request of the respective author. Due to the protection of personal data, the data are not publicly available.
